# New Appliances and Things Medical

**Published:** 1899-12-02

**Authors:** 


### NEW APPLIANCES AND THINGS MEDICAL.

### PATENT TEREBENE SOAP.

(F. S. CLEAVER, RED LION STREET, HOLBORN.)

WE have examined two varieties of this soap, namely, the household and the toilet. The soap bases are both of good quality, the toilet being superfatted and emollient. Both of them are impregnated with terebene, which being prepared by special processes from the juice of the long-leaved pine (Pinus palustris) and other of the conifera, has an exceedingly pleasant and fresh odour, somewhat resembling oil of thyme and other aromatic turpentines which are now familiar as deodorants. The qualities of the terebene, both deodorant and antiseptic, are not destroyed by the combination with a soap basis, and hence for surgical use, for the cleaning of the skin, and generally for household purposes, this soap is particularly valuable, and, unlike most antiseptic soaps, it is particularly agreeable. In chronic diseases of the skin, where a stimulating antiseptic is indicated, this soap should prove of advantage.

